# Will selenium increase lentil (*Lens culinaris* Medik) yield and seed quality?

**DOI:** 10.3389/fpls.2015.00356

**Published:** 2015-05-19

**Authors:** Dil Thavarajah, Pushparajah Thavarajah, Eric Vial, Mary Gebhardt, Craig Lacher, Shiv Kumar, Gerald F. Combs

**Affiliations:** ^1^Department of Agricultural and Environmental Sciences, Clemson University, Clemson, SCUSA; ^2^North Dakota State University, Fargo, NDUSA; ^3^Grand Forks Human Nutrition Research Center, The Agricultural Research Service/United States Department of Agriculture, Grand Forks, NDUSA; ^4^Biodiversity and Integrated Gene Management Program, International Centre for Agricultural Research in Dry Areas, RabatMorocco

**Keywords:** Lentil (*Lens culinaris*), selenium, yield increase, fertilization, biofortification, selenoproteins

## Abstract

Lentil (*Lens culinaris* Medik), a nutritious traditional pulse crop, has been experiencing a declining area of production in South East Asia, due to lower yields, and marginal soils. The objective of this study was to determine whether selenium (Se) fertilization can increase lentil yield, productivity, and seed quality (both seed Se concentration and speciation). Selenium was provided to five lentil accessions as selenate or selenite by foliar or soil application at rates of 0, 10, 20, or 30 kg Se/ha and the resulting lentil biomass, grain yield, seed Se concentration, and Se speciation was determined. Seed Se concentration was measured using inductively coupled plasma optical emission spectrometry (ICP-OES) after acid digestion. Seed Se speciation was measured using ICP-mass spectrometry with a high performance liquid chromatography (ICP-MS-LC) system. Foliar application of Se significantly increased lentil biomass (5586 vs. 7361 kg/ha), grain yield (1732 vs. 2468 kg /ha), and seed Se concentrations (0.8 vs. 2.4 μg/g) compared to soil application. In general, both application methods and both forms of Se increased concentrations of organic Se forms (selenocysteine and selenomethionine) in lentil seeds. Not surprisingly, the high yielding CDC Redberry had the highest levels of biomass and grain yield of all varieties evaluated. Eston, ILL505, and CDC Robin had the greatest responses to Se fertilization with respect to both grain yield, seed Se concentration and speciation; thus, use of these varieties in areas with low-Se soils might require Se fertilization to reach yield potentials.

## Introduction

Selenium (Se) is an essential element for humans, animals, and certain algae to make selenoproteins. However, no physiological Se requirement has been shown for higher plants (Pilon-Smits and Quinn, 2010). Nevertheless, some studies have demonstrated benefits of Se application with respect to the productivity of certain vascular plants (Hu et al., 2003; Hartikainen, 2005; Smrkolj et al., 2006; Turakainen, 2007; Lyons et al., 2009). For example, Se application increased Se concentration in potato tubers, tea leaves, and field pea seeds (Hu et al., 2003; Smrkolj et al., 2006; Turakainen, 2007). The metabolic basis of such effects is unclear.

Plants take up Se from the soil primarily as selenate or selenite, which they translocate and assimilate into organic forms (Lauchli, 1993; Ellis and Salt, 2003; Pilon-Smits and Quinn, 2010). In the chloroplast, adenosine-5-phosphoselenate is formed by the activation of ATP sulfurylase. Selenate is reduced to selenide to form selenocysteine (SeCys), which can then be converted into selenomethionine (SeMet), and methylated metabolites including Se-methylselenocysteine (methylSeCys), and dimethylselenide (Terry et al., 2000; Pilon-Smits and Quinn, 2010). Selenomethionine and SeCys are the major organic forms of Se found in legumes (Wu et al., 1997). Plants grown on seleniferous soils can be broadly categorized as Se-hyperaccumulators, which incorporate high concentrations of Se (100s of ppm), or Se-non-accumulators, which accumulate relatively low concentrations of Se (<50 ppm).

Lentil (*Lens culinaris* Medik) is a non-accumulator grain legume, with concentrations reaching 0.6–1.0 ppm Se when grown in the high-Se soils of western Canada and the mid-western USA (Brown and Shrift, 1982; Thavarajah et al., 2008). Notably, Se in naturally grown lentils is present as 90% of organic and 10% of inorganic Se (Thavarajah et al., 2007, 2008). Detailed analyses of lentil seeds indicate most organic Se is present as the seleno-amino acid SeMet, with small concentrations of SeCys and other seleno-oligopeptides such as gamma-glutamylselenocysteine (Thavarajah et al., 2008). International lentil samples vary significantly with respect to organic and inorganic Se forms, suggesting differences in the bioavailability of Se to human consumers (Thavarajah et al., 2011). Interestingly, a recent field study indicates that Se application increased grain yield, antioxidant activity, and seed Se concentration (Ekanayake et al., 2015). However, this study was conducted in high Se mid-western soils with high yielding lentil cultivars grown in the USA and Canada. Thus, it appears that Se-fertilization can improve Se nutrition, making lentils a good source of Se for Se-deficient populations. Globally, 30–100 M people are Se-deficient, mainly due to low concentrations of Se in commonly eaten foods (Combs, 2001; Ellis and Salt, 2003). The nutritional benefits of Se were first reported in Schwarz and Foltz (1957), since then, Se necessity in enzymes, cofactors, antioxidants, and protective pathways have been discovered (Combs, 2015). Selenium has multiple biological activities in mammals, depending on the level of Se intake. Low dietary Se intakes determine the expression of selenoenzymes, and higher intakes have been shown to have anti-tumorigenic potential; and very high Se intakes can produce adverse effects including selenosis and type 2 diabetes (Combs, 2015). The recommended tolerable upper Se intake level for adults is 400–450 μg/day (Food and Nutrition Board, 2000). Enriching lentils with Se may thus be an effective and sustainable means of increasing Se intakes (Thavarajah et al., 2008, 2011).

Vascular non-accumulator species respond metabolically to low dosage Se fertilization. Germ et al. (2005) demonstrated a significant (1.6-fold) yield increase in pumpkin (*Cucurbita pepo* L.) grown in the field after foliar Se application. In lettuce (*Lactuca sativa* L.), Se application was reported to increase shoot yield and increase energy reserves, produce structural changes in cell walls, increase tissue antioxidant capacity, confer protection of chloroplast enzymes, and increase senescence (Pennanen et al., 2002). Similarly, foliar application of Se has been found to increase grain yield, plant height, number of pods per plant, and harvest index of canola (*Brassica napus* L.; Lyons et al., 2009; Zahedi et al., 2009). Rahman et al. (2014) demonstrated that foliar application of a single dosage of Se fertilizer increased lentil seed Se concentration from 201 to 2772 μg kg^-1^; however, application of Se fertilizer did not effect on lentil grain yield (Rahman et al., 2014). In contrast, Ekanayake et al. (2015) showed application of Se at seeding and flowering increased lentil grain yield, seed Se concentration, and antioxidant levels. Pulse crops, mainly lentil, are staples in developing countries and part of the daily diets of many vegetarians. About, two–thirds of the world’s lentils are produced by smallholders in resource-poor countries where soil is deficient in Se (Thavarajah et al., 2011). Soil application of the trace element Se can increase lentil grain yield by a significant amount (Ekanayake et al., 2015) challenging the current thinking that Se is not essential for plants. Therefore, detailed control environmental studies are required to confirm the response of lentil accessions selected for international lentil breeding program with response to low dosage of Se influence on plant growth, grain yield, seed Se concentration, and seed Se speciation. Therefore, the objectives of this study were to determine the effect of Se fertilization (form, Se application method, and rate) on agronomic (biomass production and grain yield) and compositional (seed Se concentration and Se speciation) characteristics of selected lentil genotypes under controlled conditions.

## Materials and Methods

### Materials

Standards, chemicals, and high-purity solvents used for seed digestion and analysis were purchased from VWR International, Sigma Aldrich Co. (St. Louis, MO, USA), and Alfa Assar–A Johnson Matthey Company (Ward Hill, MA, USA) and used without further purification.

### Greenhouse Experiment

Lentil genotypes included three cultivars widely grown in the USA and Canada (Eston, CDC Redberry, and CDC Robin) and two lentil accessions (ILL 7537 and ILL 505). Seeds were obtained from the USDA-ARS Grain Legume Genetics and Physiology Research Unit, Washington State University, WA, USA, and were multiplied using single plants at the former Pulse Quality and Nutrition Laboratory, North Dakota State University (NDSU), Fargo, ND, USA. These lentil genotypes were chosen based on their current agricultural use in the USA and Canada, as well for the potential for follow-up genetic studies. More than 100 surface-sterilized seeds from each genotype were placed on sterile petri dishes with absorbent paper saturated with deionized water. Pre-germination was conducted in a dark wooden drawer at 22°C for 2 days until radical emergence. Three pre-germinated seeds from each genotype were sown in 6′′ plastic pots filled with ~150 g of a peat-perlite-vermiculite mixture (Sunshine Grow Mix Number 1, Sun Gro Horticulture Canada Inc., Toronto, ON, Canada) containing 10–15 μg Se/kg (equivalent to 51–76 g of Se/ha). The soil in each pot was saturated with deionized water and allowed to drain overnight before the weight was recorded. At seeding, pots were at 70% field capacity. Greenhouse conditions were as follows: day/night temperatures of 22/16°C, photosynthetically active radiation levels of 300 μmol/m^2^/s using a 16 h photoperiod, and 50–60% relative humidity. A total of 240 pots were seeded: three replicates of the five genotypes at four deferent Se fertilizer rates [0 (control), 10 (low), 20 (moderate), and 30 (high) kg of Se/ha], using two application methods [at seeding as a soil treatment and at flowering (10th node stage) as a foliar treatment] and two chemical forms (potassium selenate and potassium selenite). Blocks of 120 pots were separated for soil and foliar Se treatment, respectively.

#### Soil Se Treatment

Aqueous solutions of potassium selenate or potassium selenite were made to provide the above-mentioned application rates. At seeding, 1 mL of Se solution was added to each pot followed by 100 mL distilled, deionized water. Control and foliar treatment groups received no Se at seeding. All pots were watered to ~70% of free draining moisture content every day and 250 mL of nutrient solution without Se were added to all pots every 2 weeks, as per standard procedures for lentils at the NDSU Pulse Quality and Nutrition program. Nutrient concentrations of the all-purpose 20-20-20 fertilizer solution (Plant Products Co. Ltd., Brampton, ON, Canada) were 20% total N, 20% total P, 20% soluble K, 0.02% B, 0.05% chelated Cu, 0.1% chelated Fe, 0.05% Mo, 0.05% Zn, and 1% EDTA.

#### Foliar Se Application

Plants were allowed to grow for 43 days, by which time all had begun flowering. Each Se rate (0, 10, 20, 30 kg of Se/ha) was carefully prepared in a 1 L, hand-held sprayer and each plant received one application of ~80 mL applied within 5 s to the foliage. Control plants were treated with deionized water containing non-detectable (<0.01 ppm) concentrations of Se. During the foliar treatment, plants of the soil treatment series were covered and removed to a distant part of the greenhouse to avoid cross-treatment contamination.

#### Sample Preparation

At physiological maturity, all plants from both treatment series were hand-harvested and air-dried (40°C); biomass was recorded as dry weight (DW). Plants were then hand-threshed and the total seed weight per pot recorded. Seeds were stored at -40°C until analysis.

#### Se Analysis

Total Se concentration in lentil seeds was determined using inductively coupled plasma optical emission spectrophotometry (ICP-OES) after nitric acid-hydrogen peroxide digestion (Thavarajah et al., 2008). Finely ground seed samples (500 mg) were digested in nitric acid (70% HNO_3_) at 90°C for 1 h. Samples were then further digested with hydrogen peroxide (30%) before being diluted to 10 mL with nanopure water. Se concentrations were measured using ICP-OES (ICP-6500 Duo, Thermo Fisher Scientific, Pittsburg, PA, USA). Total Se measurements using this method were validated using National Institute of Standards and Technology (NIST) standard reference material 1573a (apple leaves; [Se] = 0.054 ± 0.003 mg kg^-1^). A homogenized laboratory reference material (CDC Redberry: Se = 400 ± 100 mg kg^-1^) was also used periodically for quality control. A calibration curve for Se concentration was produced using serial dilutions from 1 to 40 mg L^-1^. The limit of detection for this method was 10 ppt.

### Se Speciation

Seeds from control and low Se (10 kg Se/ha) treatments for both application methods and both Se forms were selected for analysis of organic Se species. Composite triplicate samples were ground to a fine powder, 250 mg of which was then mixed with 4 mL of nanopure water. Samples were digested with 10 mg of protease XIV (*Streptomyces griseus*) at 38°C for 90 min, after which they were centrifuged for 5 min (5000 *g*) and filtered through a 0.5 μm polytetrafluoroethylene (PTFE) membrane (Thavarajah et al., 2008). Selenium species were determined by high performance liquid chromatography (HPLC)/vapor generation/ICP-MS as previously described (Kuehnelt et al., 2006). A sample size of 30 μL was injected. The mobile phase flowed at 1 mL/min and was comprized of a phosphate buffer adjusted to pH 3.0 (5 mM NH_4_H_2_PO_4_ adjusted with H_3_PO_4_) with 1% NaBH_4_ and 5% HCl. Speciation of different Se forms was determined by ICP-MS (Elan DRCII, ICP-MS, Perkin Elmer Waltham, MA, USA) with a PE Series 200 HPLC fitted with micro pumps (Perkin Elmer, Waltham, MA, USA), a Waters Spherisorb 5 μm ODS2 4.6 × 250 mm column (Waters Corporation, Milford, MA, USA), and a Varian VGA-77 gas liquid separator (Agilent Technologies, Santa Clara, CA, USA; Kuehnelt et al., 2006). Standards (selenate, selenite, SeCys, SeMet, and Se-methylSeCys) were prepared at 40 ng Se mL^-1^.

### Statistical Analysis

The experiment used a completely randomized design with five lentil cultivars, three replicates per cultivar, four Se rates, two Se forms, and two Se application methods (*n* = 240). Data from replicates were combined and data error variances tested for homogeneity. For combined analysis, a mixed model analysis of variance was performed using the PROC GLM procedure of SAS version 9.3 (SAS, 2010), with genotypes, Se forms, Se rates, and application method as the class variables and replicates as a random factor. A separate analysis of variance was performed for each class variable to examine the effect of lentil genotype on biomass, seed yield, and total seed Se concentration. Means were separated by Fisher’s protected least significant difference (LSD) at *p* < 0.05. For Se speciation, data presented as a mean with SE (*n* = 8).

## Results

Combined analysis of variance showed that Se fertilization method, rate, and lentil genotype significantly affected lentil biomass, grain yield, and seed Se concentration at *P* < 0.05. Se form significantly affected lentil grain yield at *P* < 0.1 and seed Se concentration at *P* < 0.05. Interaction terms including lentil genotype, application method, rate, and forms were significant in some cases (**Table 1**). Selenium application significantly increased lentil biomass and grain yield as well as the Se concentration of the edible portion of the plant, i.e., the seed. The magnitude of the effect varied with the method and rate of Se application as well as lentil genotype (**Table 2**). Se fertilizer form did not affect lentil biomass or grain yield, but lentil seed Se concentration was significantly greater after selenate fertilization (2.2 μg/g) than after selenite fertilization (1.1 μg/g; **Table 2**). Foliar application of Se was the most effective method of Se fertilization with respect to increasing lentil biomass, grain yield, and seed Se concentration (**Table 2**). Both low (10 kg/ha) and high (30 kg/ha) rates of Se fertilization increased lentil biomass and grain yield compared to the control (**Table 2**). Consequently, low rate of Se fertilization was adequate in lentils to achieve optimum biomass (6538 kg/ha) and grain yield (2122 kg/ha). Se addition at the lowest rate (10 kg/ha) increased the seed Se concentration from 0.2 (control value) to 1.3 μg/g. As expected, CDC Redberry (a high yielding red lentil genotype) showed significantly higher biomass (10,522 kg/ha) and grain yield (2,815 kg/ha) than all other lentil genotypes and had moderate concentrations of seed Se (1.5 μg/g; **Table 2**). Overall, foliar application of Se at low rate (10 kg/ha) significantly increase lentil biomass and grain yield with adequate amounts of seed Se levels.

**Table 1:** Combined analysis of variance for lentils grown with different Se fertilizer forms, application methods, and rates under greenhouse conditions.

Source	*df*	Mean squares		
		Biomass	Grain yield	Seed Se
Se form	1	NS	**	*
Se application method	1	*	*	*
Se fertilizer rate	3	*	*	*
Replication	2	*	*	NS
Genotype	4	*	*	*
Se form × application method	1	NS	NS	**
Se form × fertilizer rate	3	*	*	*
Application method × fertilizer rate	3	*	*	*
Genotype × fertilizer rate	12	**	NS	*
Se form × genotype	4	**	NS	NS
Error	156	6	1.2	1.0

df, degree of freedom; *P < 0.05; **P < 0.1. NS, not significant.

**Table 2:** Overall effect of Se fertilizer form, application method, rate, and lentil genotype on biomass, grain yield, and seed Se concentration.

Treatments	Biomass (kg/ha)	Grain yield (kg/ha)	Seed Se (μg/g)
**Se form**			
(1) Selenate	6408 a	2035 a	2.2 a
(2) Selenite	6538 a	2122 a	1.1 b
**Se application method**			
(1) Soil	5586 b	1732 b	0.8 b
(2) Foliar	7361 a	2468 a	2.4 a
**Se fertilizer rates (kg/ha)**			
0	6062 c	2035 b	0.2 d
10	6538 ab	2122 ab	1.3 c
20	6452 b	1992 b	2.3 b
30	6885 a	2252 a	2.7 a
**Lentil genotypes**			
Eston	6408 c	2122 c	1.8 bc
ILL7537	3161 c	1299 e	2.3 a
ILL505	7578 b	2511 b	1.0 cd
CDC redberry	10522 a	2815 a	1.5 bc
CDC robin	4763 d	1689 d	1.4 cd

*Mean within the column for each variable and treatment followed by different letters are significantly different at *P* < 0.05. SE, pool SE of means calculated from ANOVA (*n* = 240).*

Concentrations of different Se forms present in lentils were affected by the form of Se added, rate (control and low), and application method. In general, both application methods and both forms of Se increased concentrations of organic Se (SeCys, SeMet, and methyl-SeCys). Selenium fertilization significantly increased SeMet concentrations in most lentil genotypes, with the highest concentration noted in ILL7537 (1484 μg/kg) and the lowest in CDC Robin (13 μg/kg; **Table 3**). Soil selenate fertilization increased SeMet levels in Eston, CDC Redberry, CDC Robin, and ILL 7537 compared to ILL505 (**Table 3**). Foliar Se fertilization was more effective with respect to assimilation of SeMet in ILL7537.

**Table 3:** Effect of soil and foliar application of selenate and selenite on Se speciation of lentils grown under greenhouse conditions.

Genotype	Se forms (μg/kg)	Soil				Foliar			
		Selenate		Selenite		Selenate		Selenite	
		Control	Low Se	Control	Low Se	Control	Low Se	Control	Low Se
Eston	Selenate	1.2 ± 0.1	0.1 ± 0.0	–	0.3 ± 0.0	0.2 ± 0.0	–	0.4 ± 0.0	–
	Selenite	–^‡^	–	0.2 ± 0.0	–	–	–	11.8 ± 1.0	–
	SeCys	–	5.4 ± 0.1	–	1.1 ± 0.1	1.1 ± 0.1	6.2 ± 0.1	–	2.8 ± 1.0
	SeMet	17 ± 1.0	240 ± 3.0	–	117 ± 2.0	–	21 ± 1.0	10 ± 1.0	357 ± 5.0
	Methyl-SeCys	1.5 ± 0.1	1.9 ± 0.1	–	0.7 ± 0.0	0.4 ± 0.0	–	0.9 ± 0.0	1.3 ± 0.0
ILL7537	Selenate	–	–	0.2 ± 0.0	0.3 ± 0.0	–	–	–	0.4 ± 0.0
	Selenite	–	–	–	–	–	–	0.4 ± 0.0	–
	SeCys	–	12.8 ± 1.0	–	–	–	10.7 ± 0.1	–	–
	SeMet	81 ± 3.0	346 ± 3.0	–	263 ± 3.0	–	1484 ± 5.0	108 ± 3.0	30 ± 1.0
	Methyl-SeCys	–	5.6 ± 0.1	–	1.2 ± 0.1	0.7 ± 0.0	9.6 ± 0.1	1.0 ± 0.0	1.4 ± 0.01
ILL505	Selenate	–^‡^	0.2 ± 0.0	7.9 ± 0.1	0.8 ± 0.0	0.7 ± 0.0	–	0.1 ± 0.0	0.6 ± 0.0
	Selenite	–	0.3 ± 0.0	–	–	–	0.9 ± 0.0	–	–
	SeCys	5 ± 0.1	5.4 ± 0.1	–	–	–	3.1 ± 0.1	0.6 ± 0.0	–
	SeMet	52 ± 3.0	38 ± 1.0	–	37 ± 2.0	12 ± 1.0	105 ± 5.0	41 ± 7.0	110 ± 10
	Methyl-SeCys	–	0.6 ± 0.1	0.8 ± 0.0	–	0.4 ± 0.0	2.7 ± 1.0	0.9 ± 0.0	–
CDC redberry	Selenate	0.2 ± 0.0	0.6 ± 0.0	0.4 ± 0.0	–	0.2 ± 0.0	0.2 ± 0.0	0.2 ± 0.0	0.5 ± 0.0
	Selenite	–	–	–	1.1 ± 0.0	0.8 ± 0.1	3.5 ± 0.1	–	–
	SeCys	–	–	–	5.6 ± 0.1	0.5 ± 0.0	5.3 ± 0.1	0.2 ± 0.0	–
	SeMet	18 ± 1.0	146 ± 10	67 ± 3.0	21 ± 2.0	23 ± 2.0	63 ± 4.0	22 ± 1.0	181 ± 5.7
	Methyl-SeCys	–	1.8 ± 0.0	2.5 ± 0.1	1.1 ± 0.0	–	4.7 ± 0.1	0.6 ± 0.1	–
CDC Robin	Selenate	–	0.2 ± 0.0	–	0.5 ± 0.0	0.4 ± 0.0	0.2 ± 0.0	0.3 ± 0.0	–
	Selenite	–	–	–	–	–	2.2 ± 0.1	3.6 ± 0.1	–
	SeCys	–	–	3.4 ± 0.1	0.8 ± 0.0	0.8 ± 0.0	5.8 ± 0.1	–	–
	SeMet	21 ± 1.0	157 ± 2.0	31 ± 5.0	13 ± 3.0	91 ± 7.0	137 ± 7.5	28 ± 2.0	104 ± 2.0
	Methyl-SeCys	0.7 ± 0.0	–	–	–	–	1.7 ± 0.0	0.7 ± 0.01	6.4 ± 0.1

*^‡^ND, not detected. SE, standard error for composite sample (*n* = 8 per variety).*

**FIGURE 1:**
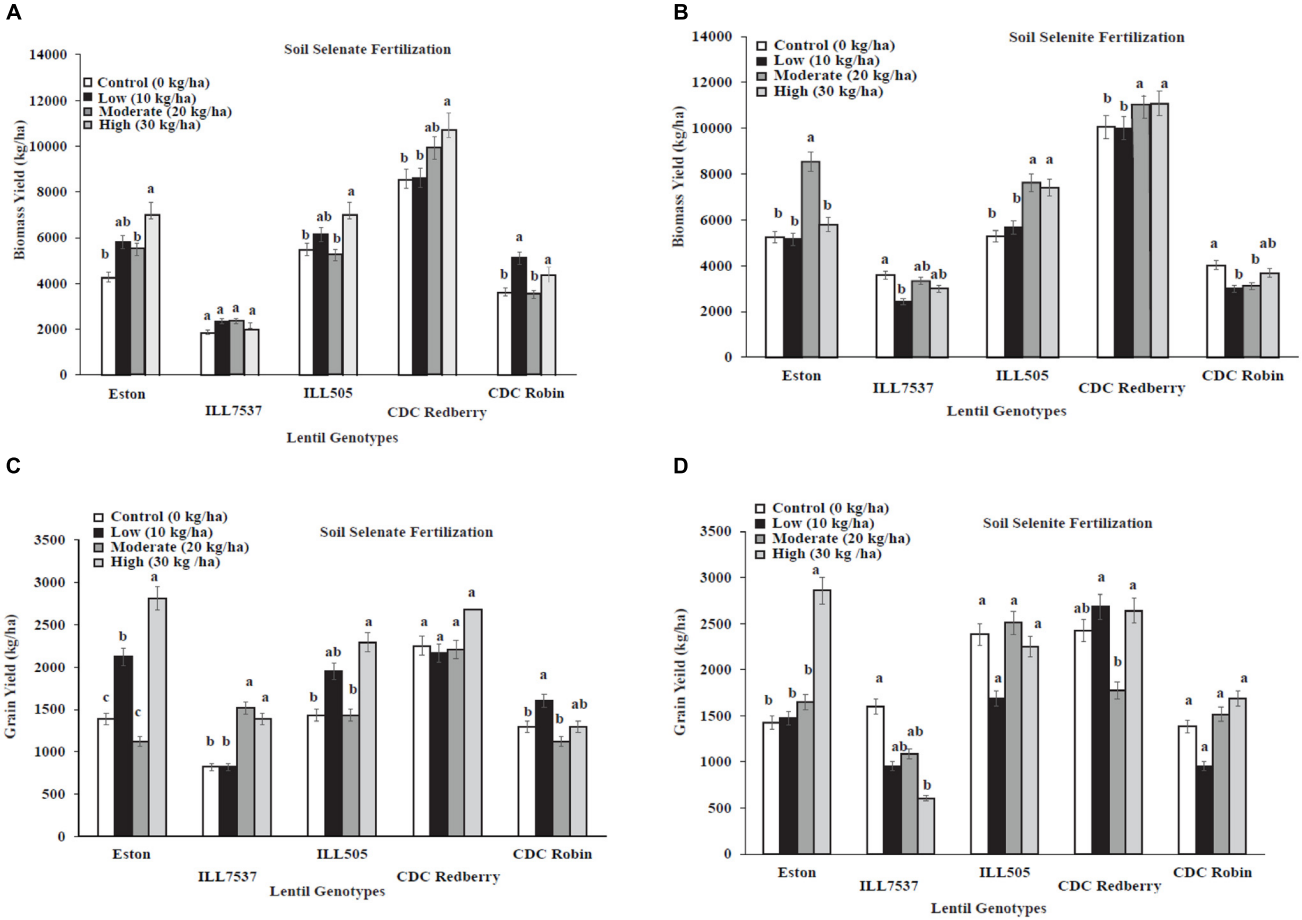
**Genotypic variation of lentil biomass yield **(A,B)** and grain yield **(C,D)** with response to soil application of selenate and selenite fertilizer at different rates**. Each genotype (bars) for each Se rate followed by different letters are significantly different at *P* < 0.05.

Response to added Se fertilizer varies with lentil genotypes. Low rate of soil selenate fertilization significantly increased biomass growth in all lentil genotypes except ILL 7537 compared to controls (**Figure 1A**). However, low rate of soil selenate significantly increased lentil grain yield in Eston and CDC Robin (**Figure 1C**). High rate of soil selenate fertilization increased yields in all genotypes except CDC Redberry grain yield. Overall, low rate of selenate fertilization was adequate to increase lentil grain yield for small seed size lentil cultivars, Eston and CDC Robin. Biomass yields of Eston, ILL505, and CDC Redberry responded positively to soil selenite fertilization at moderate and high rates (**Figure 1B**), however, soil selenite fertilization did not effect on grain yields of lentil genotypes except Eston (**Figure 1D**).

**FIGURE 2:**
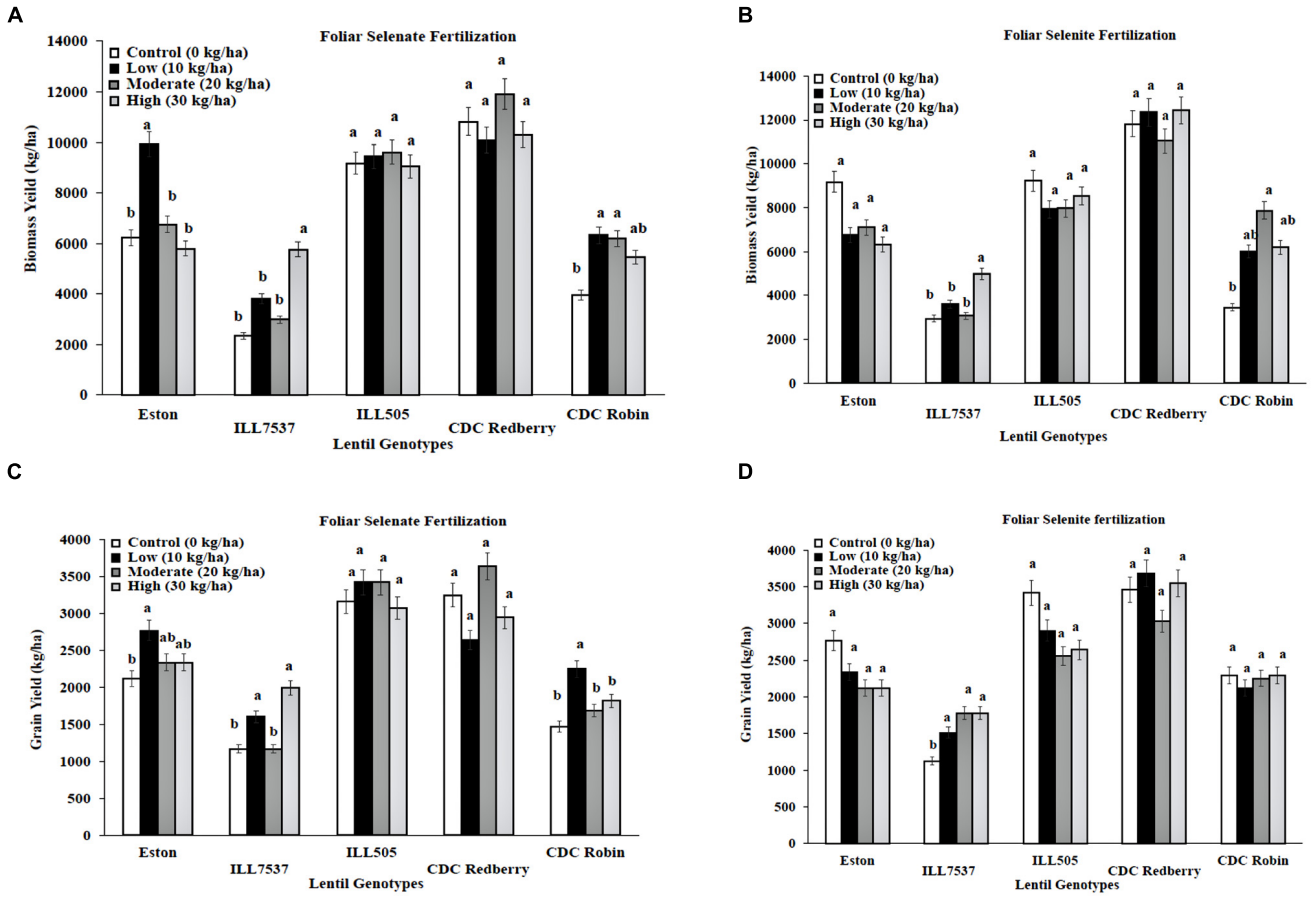
**Genotypic variation of lentil biomass yield **(A,B)** and grain yield **(C,D)** with response to foliar application of selenate and selenite fertilizer at different rates**. Each genotype (bars) for each Se rate followed by different letters are significantly different at *P* < 0.05.

Low and high rates of foliar selenate application increased biomass yield for Eston, ILL 7537, and CDC Robin compared to controls (**Figure 2A**). Low rate of foliar selenate significantly increased lentil grain yield in Eston, ILL7537, and CDC Robin (**Figure 2C**). In contrast, foliar selenite treatment did not influence biomass or grain yield for most genotypes except ILL 7537 for both yields and CDC Robin only at biomass (**Figures 2B,D**). Overall, ILL 7537 was the least responsive and Eston was the most responsive to Se fertilization.

**FIGURE 3:**
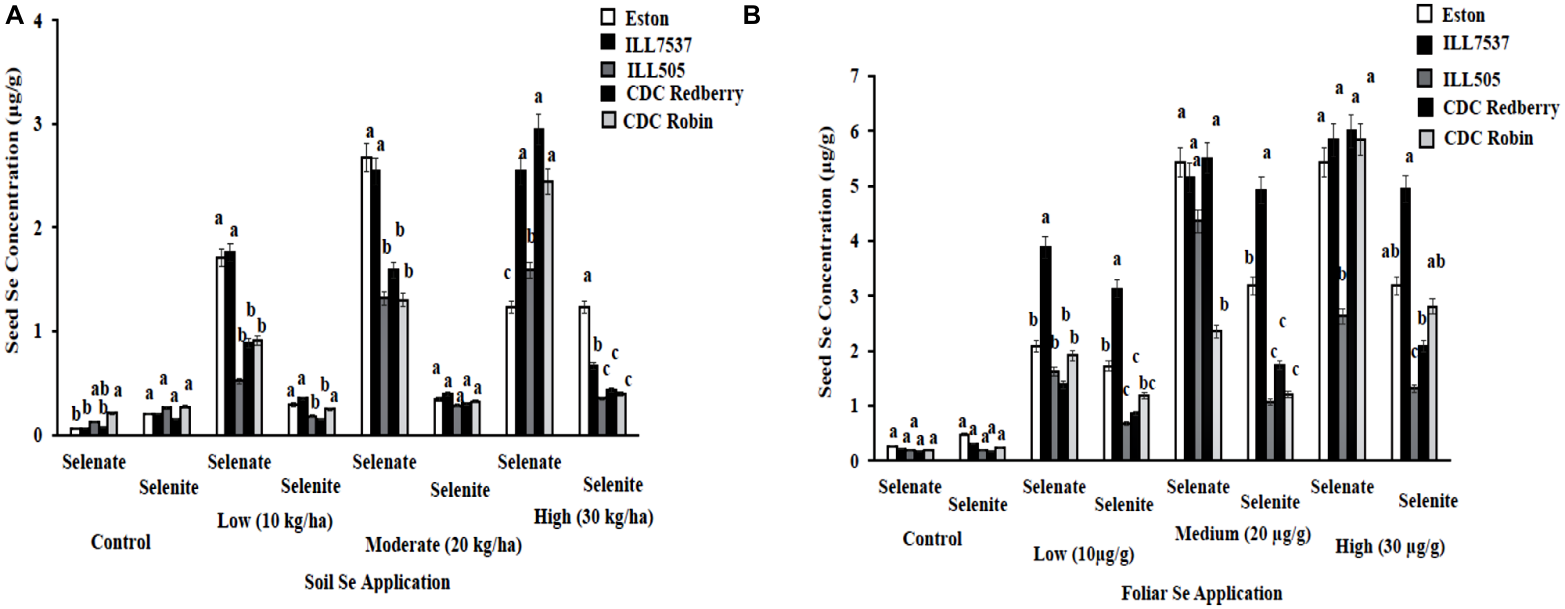
**Seed Se concentration in lentil genotypes grown under greenhouse conditions with soil application **(A)** and foliar application **(B)** of different rates and forms of Se**. Each genotype (bars) for each Se rate and form followed by different letters are significantly different at *P* < 0.05.

Lentil seed Se concentration vary with Se fertilizer form, application method, and rate. In addition, most lentil genotypes preferred selenate as a fertilizer form compared to selenite. Low and moderate rates of soil selenate significantly increased seed Se concentrations in Eston and ILL 7537 compared to other three lentil genotypes (**Figure 3A**). Eston and ILL7537 showed the most response to added Se in most cases (**Figure 3A**); CDC Redberry and CDC Robin increased seed Se concentrations with added Se, and ILL505 showed moderate responses to all Se rates. Foliar application of selenate increased the seed Se concentration in all lentil genotypes compared to selenite foliar treatment (**Figure 3B**). ILL 7537 showed the most significant response to added foliar Se at the low rate; ILL505 showed the least significant responses to all Se rates (**Figure 3B**).

## Discussion

Selenium is an essential element for mammals but has not been considered an essential element for higher plants, although benefits from Se application have been documented (Hartikainen and Xue, 1999; Turakainen et al., 2004; Lyons et al., 2009). A recent study indicated that application of selenite and selenate as a single dosage increased field grown lentil grain yield by 10 and 4%, respectively, compared to the control (Ekanayake et al., 2015). In order to investigate the impact of soil Se levels, their study should be repeated in soils with low and high levels of Se. These findings are consistent with previous research that shows crop plants respond to Se fertilization, e.g., increased tuber yield in potato (*Solanum tuberosum* L.), growth in lettuce (*Lactuca sativa* L.; Hartikainen and Xue, 1999; Turakainen et al., 2004), seed yield in mustard (*Brassica rapa* L.; Lyons et al., 2009), and lentils (Ekanayake et al., 2015). The application of Se, particularly as selenate, increased the nutritional value of lentil seed, as evidenced by the general increase in seed concentration of total Se and SeMet. This approach has been used in Finland to increase the Se content of foods and thereby the nutritional Se status of the general population (Wang et al., 1995).

Approximately, 86–95% of Se in naturally grown lentil seeds is present in organic forms, which we modeled as SeMet with a smaller amount (5–14%) as selenate (Thavarajah et al., 2007, 2008). Both forms are likely to be readily bioavailable to humans. The present findings indicate that Se fertilization can increase the amounts of Se metabolites in the seed, with responses varying among lentil cultivars. Eston and ILL7537 increased seed SeMet concentration about 4–10 folds with soil application of selenate fertilizer at a low rate. ILL 505 was the least responsive lentil cultivar to Se fertilizer for increase seed SeMet concentration. Our results clearly indicated that Eston and ILL 7537 would be the most suitable cultivars for further Se enhancement studies.

The Se concentrations of lentils produced from different parts of the world vary with soil Se level (Thavarajah et al., 2008, 2011). Lentils produced in Nepal (147–254 μg kg^-1^), Turkey (30–67 μg kg^-1^), Morocco (6–65 μg kg^-1^), and Australia (110–174 μg kg^-1^) have lower Se levels than those produced in Canada (425–673 μg kg^-1^; Combs, 2001; Thavarajah et al., 2008, 2011). Rahman et al. (2013) conducted a farmers’ lentil field survey in Bangladesh in 2010–2011 to determine the correlation between seed Se and soil Se concentration (Rahman et al., 2013). Their study indicated mean soil and lentil seed Se concentrations of 163 and 312 μg kg^-1^, respectively. Our study data, comparing added Se fertilizer rates and seed Se concentrations, suggest that the application of Se fertilizer substantially increased lentil seed Se levels (**Figures 3A,B**). Furthermore, our data clearly showed that adding Se boost lentil grain yield. Therefore, we suspect that Se fertilization can be most effective in contributing to seed Se and grain yield in low-Se soils, i.e., <5 ppm Se. Soil Se is uneven in distribution and bioavailability to plants (Combs, 2001). This suggests that soil Se concentration might play an important role in determining the final grain yield and seed Se concentration.

Lentil is one of the oldest domesticated pulse crops, originating in the Mediterranean region, Asia, and Europe in the Bronze Age. Lentil was introduced to the USA in 1916 (Baun et al., 2008). Of the five genotypes considered here, Eston, CDC Robin, and CDC Redberry are commercial genotypes with a broad range of genetic diversity for Se uptake in high Se soils (Thavarajah et al., 2008). The two selected breeding lines (ILL505 and ILL 7537) are currently used in ICARDA’s lentil breeding program for Se biofortification. Eston and CDC Robin are closely related, as CDC Robin was created from a cross between CDC Matador and Eston. Eston originated from line ILL176 found in Turkey. ILL505 originated in Germany and ILL7537 in Syria. All of these parental lines were well adapted to low Se soils. CDC Redberry is a popular high yielding genotype from the Crop Development Centre, Canada, the parental information for which is not currently available. Our findings show that Eston, CDC Robin, and ILL7537 are likely candidates for Se biofortification in low Se soils.

The physiological basis of the positive response of crop plants to Se is puzzling in view of the fact that genomic analyses of higher plants have not revealed sequences for selenoproteins (Lobanov et al., 2009). However, this view has changed after the first *de novo* assembly and annotation of a complete mitochondrial genome in American cranberry (*Vaccinium macrocarpon* Ait.; Fajardo et al., 2014). Fajardo et al. (2014) revealed the presence of two copies of tRNA-Sec and a SeCys insertion sequence element which were lost in higher plants during evolution. Therefore, the positive response to Se fertilization must involve Se metabolites. Others have noted that Se application increases antioxidant activities in plant tissues (Hartikainen, 2005; Pilon-Smits et al., 2009; Pilon-Smits and Quinn, 2010); this may enhance plant tissue growth and, subsequently, grain yield due to the redox scavenging of electrophiles, and radicals produced during photosynthesis (Cartes et al., 2005; Lyons et al., 2009).

Selenium fertilization of lentils offers the potential dual benefits of increasing both yield and nutritional value – outcomes with particular relevance to smallholder farms in low and middle-income countries. The identification of Se as a critical trace mineral and its provision through low dose fertilization may remove a barrier to the ability of smallholder farms in developing countries to achieve increased lentil yields. Such effects offer prospects for reversing the decline in lentil acreages in areas of greatest need. The resulting increase in lentil yield will benefit farmers and may contribute to reductions in malnutrition in general and micronutrient malnutrition in particular. Ultimately, progress in this direction may positively affect food security in developing countries, many of which consume 70% of their annual lentil production.

## Conflict of Interest Statement

The authors declare that the research was conducted in the absence of any commercial or financial relationships that could be construed as a potential conflict of interest.
